# Unilateral Fusion of Maxillary Lateral Incisor: Diagnosis Using Cone Beam Computed Tomography

**DOI:** 10.1155/2014/934218

**Published:** 2014-12-18

**Authors:** Iury Oliveira Castro, Carlos Estrela, Vinícius Rezende Souza, Lawrence Gonzaga Lopes, João Batista de Souza

**Affiliations:** ^1^Health Sciences Postgraduate Program, School of Medicine, Universidade Federal de Goiás, Rua 235 com 1a. Avenida, Setor Universitário, 74605-020 Goiânia, GO, Brazil; ^2^Department of Stomatologic Sciences, School of Dentistry, Universidade Federal de Goiás, Praça Universitária, Setor Universitário, 74605-220 Goiânia, GO, Brazil; ^3^Universidade Federal de Goiás, Praça Universitária, Setor Universitário, 74605-220 Goiânia, GO, Brazil; ^4^Department of Oral Prevention and Rehabilitation, School of Dentistry, Universidade Federal de Goiás, Praça Universitária, Setor Universitário, 74605-220 Goiânia, GO, Brazil

## Abstract

*Objective*. The objective of this paper is to report a dental fusion case focusing on clinical and radiographic features for the diagnosis. *Method*. To report a case of right maxillary lateral incisor fusion and a supernumerary tooth, the anatomy of the root canal and dental united portion were assessed by cone beam computed tomography (CBCT). *Results*. The clinical examination showed dental juxtaposition with the absence of interdental papilla and esthetic impairment in the right maxillary lateral incisor region. The periapical radiography did not provide enough information for the differential diagnosis due to the inherent limitations of this technique. CBCT confirmed the presence of tooth fusion. *Conclusion*. CBCT examination supports the diagnosis and provides both the identification of changes in tooth development and the visualization of their extent and limits.

## 1. Introduction

Fusion and twinning are terms commonly used to describe the clinical presentation of double teeth. The fusion process involves the tooth epithelial and mesenchymal germ layers and, as a consequence, irregular teeth formation occurs and compromises esthetics and dental alignment [1]. The union of the pulp chamber and the root canals can occur in twinning, depending on the stage of development of the junction. The differential diagnosis between the two types of anomaly is important to determine treatment [2, 3].

These anomalies are usually asymptomatic and require no treatment if they are esthetically acceptable. However, in some cases, esthetic and functional problems may appear as well as caries lesions, especially in pits and fissures, periodontal problems, asymmetries, malocclusion, and endodontic complications [4–7].

Clinical and radiographic exams may provide enough information for the differential diagnosis of fusion, twinning, and concrescence. Although periapical radiographs are routinely used to evaluate root anatomy, they might not be conclusive in some cases due to their inherent limitations [1, 4–7]. Since dental anomalies represent a three-dimensional (3D) change that may occur throughout dental surface, a careful investigation is required to obtain more accurate diagnosis and appropriate treatment.

A significant scientific revolution occurred with the advent of computed tomography (CT) [8]. This imaging study was little used in dentistry due to a number of implications, such as the cost, the amount of radiation, and the size of the equipment [9]. Scientific and technological advances in this area were made with the development of cone beam computed tomography (CBCT), which has more specific uses in different fields of dentistry [1, 10, 11].

CBCT provides 3D dental imaging, an excellent tool for more accurate evaluation and diagnosis compared with two-dimensional (2D) radiographs [12–16]. A differential feature of CBCT is the possibility of visualizing full-size images in all three planes of space, whereas 2D radiographs project the image of the structures onto a single plane, often distorted and overlapping [1, 12, 14].

The clinical applications of CBCT in dentistry have been well documented in the literature [9]. In orthodontics, CBCT has been proven useful for diagnosis of impacted teeth [17–19], detection of root resorption, alveolodental ankylosis and fracture [15, 17], assessment of bone height and volume [9, 17], investigation of temporomandibular joint and upper respiratory tract [9, 14, 19], specific determination of bone-tooth discrepancies in nonerupted teeth [17], and identification of pathologies [9, 18].

Therefore, this paper aimed to present clinical and radiographic resources for the diagnosis of dental fusion. This case report describes the clinical and conventional radiographic examinations of suspected dental fusion and the need to complement them using CBCT imaging.

## 2. Case Report

**Figure 1:**
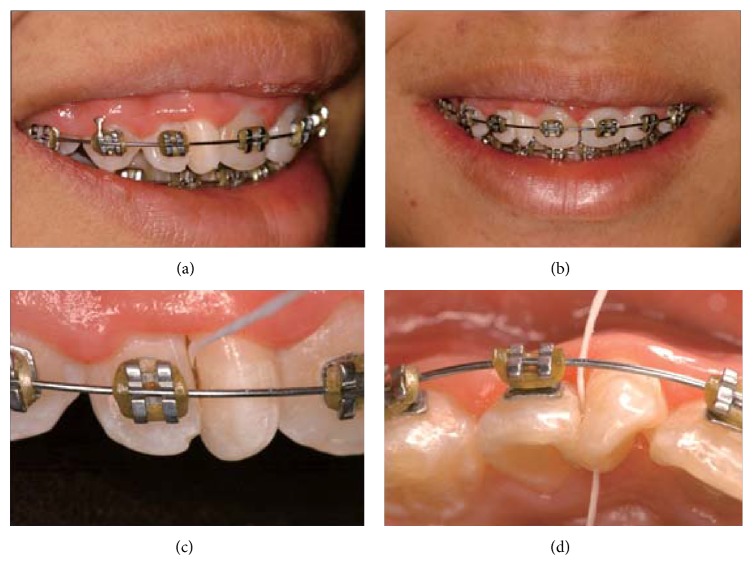
(a) Side view and (b) frontal view of the double teeth; observe the absence of interdental papilla and the midline deviation to the left. (c) Buccal view and (d) occlusal view of the double teeth with flossing to assist the diagnosis.

A 14-year-old female patient was examined at the clinic of the School of Dentistry, at the Universidade Federal de Goiás, with the main complaint of poor esthetics of the right maxillary teeth (Figures 1(a) and 1(b)). The patient had no significant past medical history and regarding her dental history the main finding was the endodontic treatment of the right maxillary central incisor. The patient presented with Angle Class I malocclusion and diastema, with upper midline deviation to the left, indicating dental asymmetry, probably as a consequence of increased mesiodistal width of the right maxillary lateral incisor.

**Figure 2:**
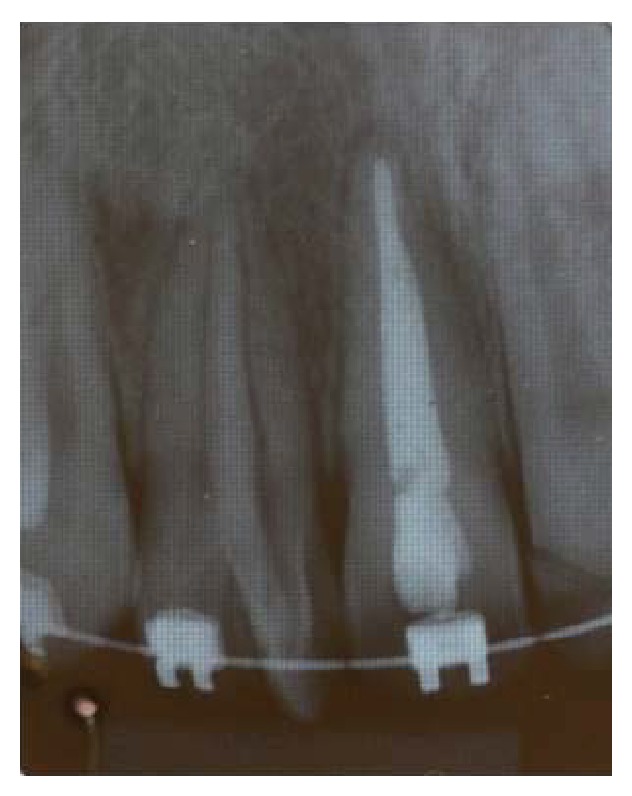
Periapical radiograph of the right maxillary lateral incisor and the supernumerary tooth with increased radiopacity in both roots suggesting overlapping image.

The clinical examination revealed that all permanent teeth had erupted, except the third and second molars. In the right maxillary lateral incisor region, two dental crowns could be seen. Flossing the interproximal region of both crowns permitted us to conclude that they were not united (Figures 1(b) and 1(c)). The caregiver reported no similar cases in the family. Since a differential diagnosis could not be offered based solely on the clinical exam, the patient underwent a periapical radiographic examination. However, this type of image did not provide enough information to elucidate the case, because it is a 2D exam that presents overlapping images (Figure 2).

**Figure 3:**
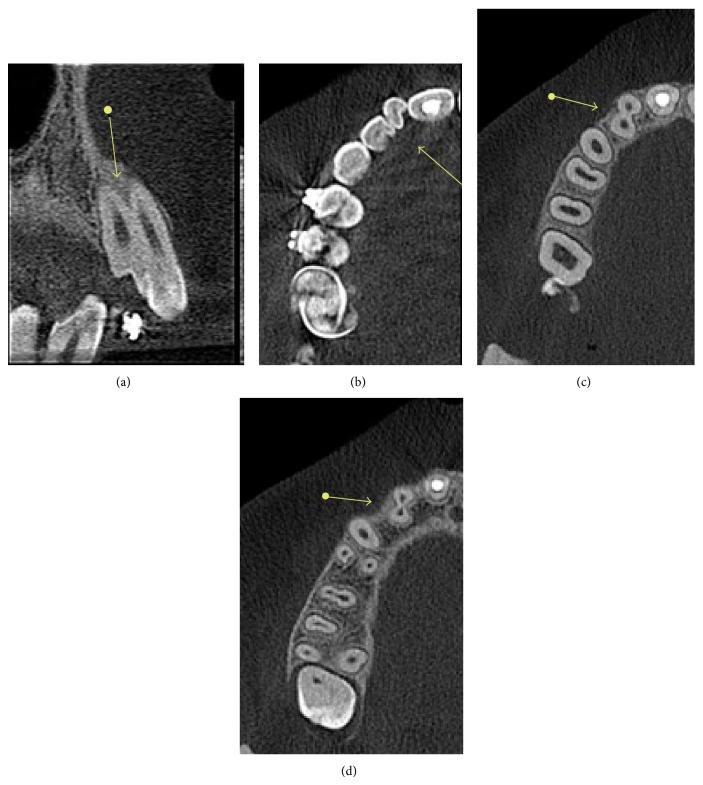
(a) Cross section of the cone beam computed tomography (CBCT) reveals the union of the right maxillary lateral incisor and the supernumerary tooth at the dentin level and shows the extent and limits of the anomaly, suggesting dental fusion. Axial sections of the CBCT show signs of fusion in the (b) cervical third, (c) middle third, and (d) apical third of the root.

Considering that the diagnosis obtained by 2D image was inconclusive and that the evaluation of the extent of the dental development anomaly was clinically impossible, a CBCT investigation was requested. The 3D exam revealed the union of the teeth at the dentin level, suggesting the differential diagnosis of dental fusion of the right maxillary lateral incisor with a supernumerary tooth (Figures 3(a), 3(b), 3(c), and 3(d)).

## 3. Discussion

Diagnostic information is essential to minimize mistakes and support decision making and appropriate planning. Accurate images allow better treatment planning and potentialize more predictable and adequate results [1, 16]. CBCT is an emerging imaging technology ideally suited for imaging the craniofacial region, including dental and maxillofacial structures. It can offer clinicians more relevant information compared with 2D radiographs [1, 13, 16–19]. In the present study, the use of CBCT was convenient and allowed the visualization of the root canals, their anatomical variations, and the portion of the double teeth that presented fusion.

Despite the extensive literature on the occurrence of double teeth, the nomenclature is still under debate. Some authors have tried to differentiate the cases by counting teeth or observing the root morphology, whereas others have used the terms fusion and twinning as synonyms. Nonetheless, there seems to be a consensus that (i) twinning is an attempt to divide a single tooth germ with the appearance of two clinical crowns supported by a single tooth root, (ii) fusion is the union of two dental germs at the enamel or dentin level, resulting in a single tooth, and (iii) concrescence is the union of two teeth at the level of cementum after root formation [3, 20–22].

The incidence of unilateral double teeth ranges from 0.4% to 2.5% in the deciduous dentition and is approximately 0.2% in the permanent dentition. The bilateral occurrence is estimated to be 0.02% in both dentitions [21, 23, 24]. There seems to be a lower incidence of double teeth in Caucasians than in Asians. The etiology remains idiopathic, but it is speculated that double teeth occur due to genetic factors, metabolic problems during dental formation, traumas, or inflammatory processes [23, 25, 26].

In some cases, the clinical and radiographic examination, as well as the simple determination of the total number of teeth in the arch, may provide enough information for the differential diagnosis between fusion, twinning, and concrescence [6]. However, the distinction between twinning and fusion by counting teeth is unreliable, because the anomaly can occur between a normal tooth and a supernumerary tooth as in the present study [27–29].

The use of 3D images provides a better view of the teeth and guides the choice of the appropriate treatment. The treatment can be performed with a surgery (extraction, sectioning, or extraction after sectioning and immediate reimplantation) [7] or, more conservatively, with reduction of mesiodistal dimensions to preserve the pulp and avoid prostheses [4, 30]. The use of orthodontic appliances may also be necessary for correction of esthetics and functional problems caused by the dental anomaly [7].

In the present case, clinical examination and conventional 2D radiographs were insufficient for the differential diagnosis and, consequently, for assessing the anatomical extent of the dental development anomaly. Therefore, the indication of CBCT scan was justified and this 3D diagnostic tool was essential for suitable orthodontic treatment planning and execution.

## 4. Conclusion

CBCT imaging not only supported the differential diagnosis and the identification of changes in tooth development, but also allowed the visualization of their extent and limits.
